# Knowledge and attitude toward geriatric nursing care and associated factors among nurses working at hospitals in Hawassa City, Ethiopia, 2022

**DOI:** 10.3389/fmed.2024.1284845

**Published:** 2024-06-07

**Authors:** Workineh Abera, Alemneh Kabeta Daba, Bereket Beyene Gebre, Mathewos Ashagere, Tomas Yeheyis, Dereje Addisu, Tsegahun Amlaku, Fikru Tadesse, Tinbete Samuel

**Affiliations:** ^1^Department of Nursing, College of Medicine and Health Science, Wachemo University, Durame, Ethiopia; ^2^School of Nursing, College of Medicine and Health Sciences, Hawassa University, Hawassa, Ethiopia

**Keywords:** attitude, Ethiopia, geriatric nursing care, Hawassa, knowledge, nurses

## Abstract

**Background:**

Globally, the fastest growth in the number of older people combined with chronic and age-related medical conditions experienced by the older adult placed great demand on geriatric care. Thus, nurses are required to be knowledgeable and have a desirable attitude toward geriatric nursing care. Therefore, this study aimed to assess knowledge and attitude toward geriatric nursing care and associated factors among nurses working at hospitals in Hawassa City, Ethiopia.

**Methods:**

Hospital-based cross-sectional study was conducted from June 30 to July 30, 2022, among 365 nurses. The hospitals and study participants were selected by using purposive and simple random sampling methods, respectively. Data were collected using self-administered questionnaires. Descriptive statistics were computed to generate descriptive results. Binary and multivariable logistic regressions were used to identify predictors at *p*-value <0.05.

**Results:**

About 39.2% of nurses had good knowledge and 49.3% of the nurses showed a positive attitude toward geriatric nursing care. Nurses with BSc degree or above [AOR 2.5, 95% CI, (1.2–5.6)], having lived with older people [AOR 2.2, 95% CI, (1.4–3.6)], nurses with 6–10 years [AOR, 2.8, 95% CI, (1.4–5.57)] and >10 years of work experience [AOR 4.2, 95% CI, (1.6–10.8)] were more likely to have knowledge about geriatric nursing care. Having BSc degree or above [AOR 2.7, 95% C.I, (1.2–6)], 6–10 years [AOR 3, 95% CI, (1.48–6.3)], and >10 years [AOR 3.9, 95% CI, (1.4–10.99)] of work experience, living experience with older people [AOR 1.7, 95% C.I:1.05–2.7], knowledge about geriatric care [AOR 3, 95% CI,(1.85–4.92)], and having worked at adequate space [AOR 1.7, 95% CI: 1.009–2.8] increased odds of good attitude toward geriatric nursing care.

**Conclusion:**

Less than half of nurses demonstrated good knowledge and positive attitude toward geriatric nursing care. Higher level of education, higher work experience, living with older people were significantly associated with knowledge and attitude toward geriatric nursing care. Additionally, working in an adequate space and having knowledge about geriatric care positively influenced attitude toward geriatric nursing care. Therefore, nursing schools and hospitals should conduct training and consider incorporating more content about geriatric care into nursing education to enhance nurses’ knowledge and attitude.

## 1 Introduction

Geriatric nursing care refers to the care and services provided to older people (1). According to the United Nations definition, older people are individuals who are 60 years of age or older (2). Geriatric care is an emerging context that is in great demand due to a current demographic shift brought on by an increase in life expectancy (3, 4).

Globally the number of geriatric people aged 60 years or older in the population is increasing at the fastest pace. By 2050, 2.1 billion (one out of every five persons) will be 60 years or older, with 80% of them will be living in low- and middle-income nations (5). A scoping review of the literature about nurses’ preparedness in low and middle-income countries found that, in the majority of studies, nurses had moderate to inadequate knowledge about geriatric nursing care (6). Africa’s older adult population is projected to more than triple in the next decades, nearing that of Europe by 2050, with Sub-Saharan Africa which includes Ethiopia accounting for the largest share of the continent’s older adult population (7). Following this global trend of population aging the demand for professionals in geriatric nursing care is increasing (8).

Despite the demand created by an increase in their number, older people have unique and varied entities that call for a wide range of healthcare providers to meet their needs. This requires specific skills from nursing as well as knowledge of the biological, psychological, social, and cultural theories that pervade aging (3). As a result, geriatric care is becoming more popular as a nursing specialty, and it requires unique knowledge and skills (9). Like the rest of the world, Ethiopia has a growing older adult population (7), but chronic and age-related disorders that necessitate skilled nursing care pose a greater threat to their health. In Ethiopia, about 75% of the old age people were suffering from at least one chronic disease. Eye problems (29%), arthritis (20.17%), and hypertension (11.83%) are the three most common problems followed by urinary tract, hearing, and heart conditions that older people are seeking medical treatment for and 23% of older people were not taking medical treatment (10).

However, due to a shortage of skilled professionals and well-organized geriatric care units, and a lack of geriatric care training institutions in the country, the care of older people is not well addressed (11). According to a study conducted in two older adult homes in Ethiopia, formal caregivers from both government and non-government long-term care settings lacked any geriatric qualifications and training (12). A few studies done elsewhere in the country showed that the majority of nurses in public hospitals had poor knowledge and negative attitude toward geriatric nursing care (11, 13). Although the health care system is not well prepared for the aged, the need for geriatric nursing care is rising in Ethiopia as the population ages and the accompanying age-related and chronic medical conditions experienced by the older adult. As a result, nurses as key health care providers are required to be prepared with better knowledge and a positive attitude toward geriatric nursing care. However, evidence is limited on nurses’ knowledge and attitude toward geriatric nursing care in Ethiopia, particularly in the study area. Therefore, the purpose of this study was to assess knowledge and attitude toward geriatric nursing care and identify associated factors among nurses working at hospitals in Hawassa city, Ethiopia. The findings of this study will serve as a basis for the development of intervention strategies aimed at enhancing nurses’ knowledge and attitude toward geriatric nursing care, which will eventually help to improve the quality of care given to the older adult.

## 2 Methods and materials

### 2.1 Study area, period, and design

The study was conducted at selected hospitals in Hawassa city from June 30, 2022, to July 30, 2022, G.C. Hawassa is a city in Ethiopia, in the Great Rift Valley and it is located 273 km south of Addis Ababa via Bishoftu. In Hawassa city, there are four public hospitals: Hawassa University Comprehensive Specialized Hospital (HUCSH), Adare General Hospital, Motite Fura Primary Hospital, and Tula Primary Hospital along with six private hospitals namely: Abem Primary Hospital, Alatiyon General Hospital, BeteAbreham Primary Hospital, Kibru Primary Hospital, Naol Primary Hospital, and Yanet Primary Hospital. A Hospital-based cross-sectional study was conducted among nurses working at hospitals in Hawassa city.

### 2.2 Source and study populations

All nurses who were working at hospitals in Hawassa city were the source population and the sampled nurses working at the selected hospitals in Hawassa city were the study population.

Nurses specialized in Pediatrics nursing and Neonatology, and nurses working part-time in private hospitals were excluded from the study because some nurses in public hospitals also work part-time in private hospitals in Hawassa city.

### 2.3 Sample size and sampling technique

The sample size was determined by using the formula for single population proportion by considering assumptions like; a 95% confidence level, and 5% margin of error, for the first and second objectives and double population proportion formula for the third and fourth objectives. The largest sample size was selected as shown below


$$\frac{n={\left(Z\;a/2\right)}^{2}p\;\left(1-p\right)}{d^{2}}$$

*p* = 29% (11).


$$n=\frac{{\left(1.96\right)}^{2}0.29\left(1-0.29\right)}{{\left(0.05\right)}^{²}}=316$$

The total number of nurses in selected hospitals were 709. The population correction formula; n/ (1+ [n/N]), was used since the source population was less than 10,000. Then, 316/ (1 + 316/709) =226. By using design effect (1.5) the sample size is 1.5*226 = 339, then adding 10% non-response rate final sample size = 373.

The study hospitals were selected by using a purposive sampling method based on the number of nurses they have. From the total of 10 hospitals in Hawassa; 3 were included in the study. Two public hospitals (Hawassa University Comprehensive Specialized Hospital (HUCSH) and Adare general hospital) and 1 private hospital (Alatiyon general hospital) were selected. The sample size was allocated proportionally to each hospital based on the number of nurses and study participants were selected by simple random sampling method by using the list of nurses as a sampling frame. Total number of Nurses (*N*) =709. i.e., (HUCSH = 496, Adare general hospital = 175, Alatiyon general hospital = 38). The sample size was allocated proportionally for each hospital.

1. HUCSH = 373*496/709 = 261.
2. Adare General Hospital = 373*175/709 = 92.
3. Alatiyon General Hospital = 373*38/709 = 20.

### 2.4 Study variables

Knowledge and attitude toward geriatric nursing care were dependent variables of the study. Socio-demographic variables such as; Age, Educational status, sex, living with older people, work experience, marital status, and institutional and professional related factors such as; hospital type, communication and adequacy of working room were independent variables of the study.

### 2.5 Data Research Topic tool and procedures

The data Research Topic process was facilitated by 3 BSc nurses from an unstudied area and supervised by one experienced nurse. Data were collected by using a self-administered questionnaire. The questionnaire has four sections: the first part was about socio-demographic data, Part two: the knowledge about Older Patients-Quiz (KOP-Q) scale was adopted from the previous study, to collect data about nurses’ knowledge, the tool is originally validated and developed in the Netherlands and by Jorean Dikken (11, 14). Part three: the older People in Acute Care Survey (OPACS) scale was adopted from a previous study in Ethiopia to collect data about nurses’ attitude and the tool was originally developed by Jorean Dikken and validated in the United States (11, 15) and part four was about personal and institutional related factors.

### 2.6 Data quality assurance

One day training was given to data collectors about facilitation of the data Research Topic process. The questionnaire was pretested on 5% (18 nurses) at Durame general hospital before the actual data Research Topic time and necessary corrections and modifications were made. The reliability of tools in terms of internal consistency was checked by using Cronbach’s alpha and it was 0.73 for the knowledge scale and 0.87 for the attitude scale. The completeness of data was checked during data Research Topic and before analysis.

### 2.7 Data processing and analysis

The data were entered into epi data version 4.6 and cleaned, checked for completeness and accuracy, then exported to SPSS version 26 for analysis. Descriptive statistics were displayed in tables and graphs, mean, percent, and frequency were calculated and summarized in the text. The knowledge assessment part consists of 30 true/false questions. These questions were recoded into correct and incorrect during analysis. The correct answer was coded as 1 and the incorrect coded as 0. Finally, respondents who scored 75% and above (23 correct answers and above) on KOP-Q were categorized as having good knowledge and who scored below 23 correct answers were categorized as having poor knowledge about geriatric nursing care. The attitude was assessed by using the OPACS attitude measurement scale which consists of 34 items arranged in a five-point Likert scale ranging from strongly disagree to strongly agree, the responses were dichotomized into positive and negative based on a mean score of 3 for each respondent after reverse scoring negative questions during analysis. Model fitness was checked by using Hosmer and Lemeshow model and the results were 0.78 for knowledge and 0.54 for attitude. Binary logistic regression was carried out to identify the variables eligible for multivariable analysis. Variables with a *p*-value less than 0.25 in binary logistic regression were fitted into the multivariable logistic regression model (16). In multivariable logistic regression, the variables are considered significant at *p*-value <0.05.

## 3 Results

### 3.1 Socio demographic characteristics

A total of 365 nurses responded to the data inquiry which made the response rate 97.8%. Half (50.1%) of the respondents were in 20–29 years of age range. More than half of the respondents 193 (52.9%) and 213 (58.4%) were found to be female and married, respectively. The majority 322 (88.2%) of the respondents were BSc degree holders in nursing. Nearly half 181 (49.6%) of the respondents practiced nursing for 1–5 years. Concerning living with the older adult; more than half of the respondents 194 (53.2%) reported that they ever lived with older people (Table 1).

**Table 1:** Socio demographic characteristics of nurses working at selected hospitals in Hawassa city, Ethiopia, 2022 (*n* = 365).

Socio demographic characteristics		Frequency	Percent
Sex	Male	172	47.1
	Female	193	52.9
Age	20–29	183	50.1
	30–39	160	43.8
	≥40	22	6.0
Marital status	Single	152	41.6
	Married	213	58.4
Level of education	Diploma	43	11.8
	BSc or above	322	88.2
Lived with older people	Yes	194	53.2
	No	171	46.8
Year of experience in nursing	≤ 5	181	49.6
	6–10	124	34.0
	>10	60	16.4

### 3.2 Personal and institutional factors

More than two-thirds (70.1%) of the respondents were practicing nursing in the teaching hospital. About 69% of the respondents perceive that the space is not adequate for nursing practice. More than two-thirds (67.4%) of respondents reported that they like to communicate with older people and their families during caregiving.

### 3.3 Knowledge and attitude of nurses toward geriatric nursing care

**Figure 1:**
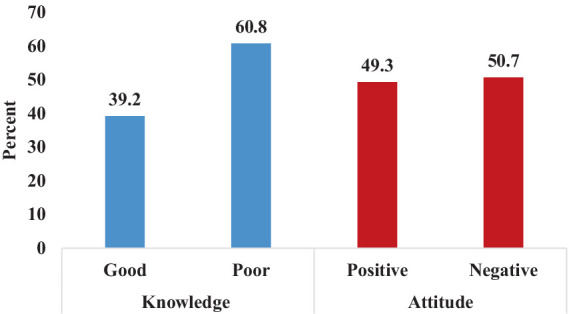
Knowledge and attitude toward geriatric nursing care among nurses working at selected hospitals in Hawassa City, Ethiopia, (*n* = 365).

Overall; 143(39.2%) (95% C.I, 34–44%) of nurses have good knowledge about geriatric nursing care and 180(49.3%) (95% C.I, 44–54%) of nurses have a positive attitude toward geriatric nursing care (Figure 1; Tables 2, 3).

**Table 2:** Frequency distribution of knowledge test questions (KOP-Q) among nurses working at selected hospitals in Hawassa city, Ethiopia, 2022 (*n* = 365).

	Item	Correct	Incorrect
201	Forgetfulness, concentration issues and indecisiveness are parts of aging rather than indicators of depression	204 (55.9%)	161 (44.1%)
202	Urinary incontinence in an older person may indicate that the person is suffering from a urinary tract infection	292 (80%)	73 (20%)
203	Patients with a cognitive disorder, such as dementia, are at increased risk for delirium	311 (85.2%)	54 (14.8%)
204	Malnutrition can have negative effects on thinking and observation skills	330 (90.4%)	35 (9.6%)
205	In general, older people are more sensitive to medication because their kidney and liver functions are declining	299 (81.9%)	66 (18.1%)
206	Meeting with families during patient assessment is only required for persons suffering from dementia	280 (76.7%)	85 (23.3%)
207	For older people, bed rest is important to enhance recovery	201 (55%)	164 (45%)
208	Patients rarely remember that they were anxious and/or restless during delirium	183 (50.1%)	182 (49.9%)
209	Older people need less fluid because they exercise less	215 (59%)	150 (41%)
210	Asking patients whether they have fallen in the past 6 months is a good way of assessing increased risk of falling	285 (78%)	80 (22%)
211	Pressure that cuts off the blood supply to tissue for two hours may result in pressure ulcers	256 (70%)	109 (30%)
212	Depression is recognized in older people less frequently than it is in younger people	276 (75.6%)	89 (24.4%)
213	Lowering the frequency of a medication is an effective intervention to achieve adherence by patients	253 (69.3%)	112 (30.7%)
214	Incontinent patients must have their soiled clothing changed but do not need to be placed on the toilet afterwards	240 (66%)	125 (34%)
215	It is good to have older people drink more often, because they have a reduced thirst sensation	271 (74.2%)	94 (25.8%)
216	In the case of delirium, bright lighting should be used to illuminate all of the corners of the room	209 (57%)	156 (43%)
217	Medication may cause geriatric problems such as memory deficits, incontinence, falling and depression	237 (65%)	128 (35%)
218	Overburdening of family caregivers may lead to abuse of the person for whom they are providing care	302 (82.7%)	63 (17.3%)
219	It is good to provide extensive instruction about how to complete tasks to patients suffering from apraxia	195 (53%)	170 (47%)
220	When speaking to hearing-impaired older patients, it is best to speak at normal volume	237 (65%)	128 (35%)
221	An older person with a BMI of >25 cannot be undernourished	233 (64%)	132 (36%)
222	In the case of difficulty swallowing, all medicines must be ground to ensure that patients ingest them	221 (60.5%)	144 (39.5%)
223	In the case of depression, memory problems may occur	297 (81.4%)	68 (18.6%)
224	Most family caregivers do not need additional support from homecare services	266 (72.9%)	99 (27.1%)
225	As a nurse, you have to speak clearly into the ear of the hearing-impaired older Patient	192 (52.6%)	173 (47.4%)
226	Pain medication should be administered to older people as little as possible, due to the possibility of addiction	209 (57.3%)	156 (42.7%)
227	We identify pressure ulcers only if blister formation or abrasions have occurred	232 (63.6%)	133 (36.4%)
228	In the case of delirium, activities should be spread out evenly over the day	230 (63%)	135 (37%)
229	The risk of falling is higher for people in the hospital setting compared with those who are living at home	239 (65.5%)	126 (34.5%)
230	Stress incontinence may occur in patients who are not capable of opening their own trousers	195 (53.4%)	170 (46.6%)

**Table 3:** The frequency distribution of OPACS scores among nurses working at selected hospitals in Hawassa city, Ethiopia, 2022 (*n* = 365).

SN	Items	Positive	Negative
301	Do you like to care for older patients	284 (77.8%)	81 (22.2%)
302	*Do you agree older patients are confused	171 (46.8%)	194 (53.2%)
303	*Do you agree older patients pretend not to hear you	166 (45.5%)	199 (54.5%)
304	*Older patients are a nuisance to care for	157 (43%)	208 (57%)
305	*Older patients are more likely to be depressed than younger patients	113 (31%)	252 (69%)
306	*Older patients have to follow special diets	81 (22.2%)	284 (77.8%)
307	*Older patients do not know the actions and interactions of their medications	113 (31%)	252 (69%)
308	*Older patients require less pain relieving medication than younger patients	128 (35.1%)	237 (64.9%)
309	*Older patients become addicted to sleeping medications easily	114 (31.2%)	251 (68.8%)
310	*Incontinent patients are bothersome	110 (30.1%)	255 (69.9%)
311	*Urinary incontinence is part of the aging process	98 (26.8%)	267 (73.2%)
312	Older patients are more concerned with their bowel habits than younger patients	200 (54.8%)	165 (45.2%)
313	Older patients are embarrassed when their bodies are exposed	208 (57%)	157 (43%)
314	*Too many older patients receive life-sustaining treatment	90 (24.7%)	275 (75.3%)
315	Older patients have more discharge problems than do younger patients	195 (53.4%)	170 (46.6%)
316	At the time of discharge older patients are likely to be more dependent than younger patients	242 (66.3%)	123 (33.7%)
317	Older patients require placement in long term care following hospital admission	207 (56.7%)	158 (43.3%)
318	*Older patients have extensive lengths of stay and take up beds that could be used for sicker patients	118 (32.3%)	247 (67.7%)
319	*There are too many older patients in acute care hospitals	138 (37.8%)	227 (62.2%)
320	It would be a good idea for all hospitals to have an acute geriatric unit	260 (71.2%)	105 (28.8%)
321	Older patients are likely to be on more medication when admitted to the hospital than younger patients	168 (46%)	197 (54%)
322	Older patients become confused in a new setting	172 (47.1%)	193 (52.9%)
323	Older patients feel isolated in the acute care setting	158 (43.3%)	207 (56.7%)
324	*In the hospital, eating and drinking are the most common activities performed by older patients	151 (41.4%)	214 (58.6%)
325	Older patients have more skin problems than younger patients	216 (59.2%)	149 (40.8%)
326	Older patients are more likely to require assistance with mobility than younger patients	263 (72.1%)	102 (27.9%)
327	A lot of older patients have stiff joints	239 (65.5%)	126 (34.5%)
328	Older patients tend not to tell health professional if they are incontinent	199 (54.5%)	166 (45.5%)
329	Older patients experience changes in bowel elimination patterns in the acute care setting	176 (48.2%)	189 (51.8%)
330	Older patients are more likely to have open surgical procedures than laparoscopic surgery	156 (42.7%)	209 (57.3%)
331	Older patients become confused after operations/procedures	225 (61.6%)	140 (38.4%)
332	Older patients are more likely to develop post-operative complications	220 (60.3%)	145 (39.7%)
333	Older patients are particularly prone to nosocomial infections	220 (60.3%)	145 (39.7%)
334	Early discharge is difficult to achieve with older patients	235 (64.4%)	130 (35.6%)

^*^Negative statements.

### 3.4 Factors associated with knowledge about geriatric nursing care

On binary logistic regression variables with a *p*-value, of less than 0.25 were selected for further analysis in multivariable logistic regression. In multivariable logistic regression; level of education, year of work experience, and living with older people 60 years and above were significantly associated with knowledge about geriatric nursing care at *p*-value <0.05.

Study participants with a BSc degree or above in nursing were two and half times [AOR 2.5, 95% CI, (1.2–5.6)] more likely to have good knowledge about geriatric nursing care than study participants with a diploma in nursing. Nurses having lived with older people aged 60 years or above were more than two times [AOR 2.2,95% CI, (1.4–3.6)] more likely to have good knowledge about geriatric nursing care than nurses who did not live with older people aged 60 years or above. Nurses who have 6–10 years of experience in the nursing profession were nearly three times [AOR, 2.8, 95% CI, (1.4–5.57)] more likely to have good knowledge about geriatric nursing care than nurses with ≤5 years of experience in the nursing profession and nurses who have more than 10 years of professional experience were more than four times [AOR 4.2, 95% CI, (1.6–10.8)] more likely to have good knowledge about geriatric nursing care than nurses who have ≤5 years of work experience in the nursing profession (Table 4).

**Table 4:** Binary and multivariable analysis of knowledge about geriatric nursing care with associated variables in selected hospitals at Hawassa city, Ethiopia, 2022 (*n* = 365).

Variables		Knowledge		COR (95% CI)	AOR (95% CI)	*p* value
		Good (143)	Poor (222)			
Sex	Female	81	112	1.28 (0.84–1.95)	1.3 (0.8–2.09)	0.228
	Male	62	110	1	1	
Age	20–29	60	123	1	1	
	30–39	66	94	1.4 (0.9–2.2)	0.51 (0.25–1.03)	0.06
	≥40	17	5	6.97 (2.4–19.8)*	1.36 (0.34–5.4)	0.657
Level of education	Diploma	10	33	1	1	
	BSc or above	133	189	2.3 (1.1–4.9)*	2.5 (1.17–5.57)*	0.018
Lived with older people	Yes	95	99	2.4 (1.6–3.8)*	2.2 (1.4–3.6)*	0.001
	No	48	123	1	1	
Years of experience in nursing	≤ 5	52	129	1	1	
	6–10	54	70	1.9 (1.2–3.09)*	2.8 (1.4–5.6)*	0.003
	>10	37	23	3.99 (2.16–7.36)*	4.2 (1.6–10.8)*	0.003

^*^Significant at *p*-value < 0.05.

### 3.5 Factors associated with attitude toward geriatric nursing care

On binary logistic regression variables with a *p*-value, of less than 0.25 were selected for further analysis in multivariable logistic regression. In multivariable analysis; level of education, year of work experience in nursing, living experience with older people age 60 years or above, adequacy of workspace/room during caregiving, and knowledge about geriatric nursing care were significantly associated with attitude toward geriatric nursing care at *p*-value <0.05.

Nurses having a BSc degree or above in nursing were about three times [AOR 2.7, 95% C.I. (1.2–6)] more likely to have a positive attitude toward geriatric nursing care than nurses who have a diploma in nursing. Respondents who had lived with older people with age 60 years or above were about two times [AOR 1.7, 95% C.I. (1.05–2.74)] more likely to have a positive attitude toward geriatric nursing care than those who did not live with older people with age of 60 years or above. Regarding years of work experience of the respondents; nurses with 6–10 years of experience in the nursing profession were three times [AOR 3, 95% CI, (1.48–6.3)] more likely to have a positive attitude towards geriatric nursing care than nurses who have ≤5 years of experience, and nurses who have more than 10 years of professional experience were about four times [AOR 3.9, 95% CI, (1.4–10.99)] more likely to have a positive attitude toward geriatric nursing care than nurses who have ≤5 years of work experience in the nursing profession. Nurses who perceived that the workspace/room was adequate during caregiving for older people were almost two times [AOR 1.7, 95% CI, (1.009–2.8)] more likely to have a positive attitude toward geriatric nursing care than nurses who did not work at adequate space and nurses having good knowledge about geriatric nursing care were three times [AOR 3, 95% CI, (1.85–4.92)] more likely to have a positive attitude toward geriatric nursing care than nurses having poor knowledge about geriatric nursing care (Table 5).

**Table 5:** Binary and multivariable analysis of attitude toward geriatric nursing care and associated factors among nurses working in selected hospitals in Hawassa city, Ethiopia, 2022 (*n* = 365).

Variables		Attitude		COR (95% CI)	AOR (95% CI)	*p* value
		Positive (180)	Negative (185)			
Age	20–29	77	106	1	1	
	30–39	86	74	1.6 (1.04–2.4)*	0.53 (0.25–1.1)	0.094
	≥40	17	5	4.7 (1.6–13.2)*	0.6 (0.14–2.7)	0.53
Marital status	Married	112	101	1.37 (0.9–2)	0.9 (0.5–1.59)	0.74
	Single	68	84	1	1	
Level of education	BSc or above	169	153	3.2 (1.6–6.6)*	2.7 (1.2–6)*	0.015
	Diploma	11	32	1	1	
Lived with older people	Yes	113	81	2.1 (1.4–3.3)*	1.7 (1.05–2.74)*	0.030
	No	67	104	1	1	
Years of experience in nursing	≤ 5	67	114	1	1	
	6–10	71	53	2.28 (1.43–3.6)*	3 (1.48–6.3)*	0.002
	>10	42	18	3.97 (2.1–7.4)*	3.9 (1.4–10.99)*	0.009
Worked at adequate space	Yes	69	43	2 (1.3–3.23)*	1.7 (1.009–2.8)*	0.046
	No	111	142	1	1	
Like to communicate with older/family	Yes	138	108	2.3 (1.49–3.68)*	1.6 (0.99–2.76)	0.054
	No	42	77	1		
Knowledge	Good	100	43	4 (2.6–6.47)*	3 (1.85–4.92)*	0.000
	Poor	80	142	1		

^*^Significant at *p*-value < 0.05.

## 4 Discussion

There is a high need for geriatric nursing care due to the rapidly growing older population with age-related and chronic medical conditions. Nurses play a crucial role in providing these services, requiring well-prepared nurses with good attitudes and knowledge.

The current study found that 39.2% (95% C.I: 34–44%) of nurses have good knowledge about geriatric nursing care. The finding was consistent with the studies in Bahir Dar Ethiopia (42.7%) (17) and west Shoa (37.2%) (13). This finding was lower than the previous studies conducted in Saudi Arabia (65%) (18), Taiwan Teaching Hospital (55.76%) (19), Israel (51%) (20), teaching hospital in Ghana (88.7%) (21), and study among critical care nurses in Egypt (79.8%) (22). This disparity could be attributed to difference, in study settings, as well as lack of training and formal education in the study area about geriatric nursing care. Because the aforementioned studies were conducted in the countries where the nurses had gerontology educational backgrounds and trainings.

The finding of the current study revealed 49.3% (95% C.I: 44–54%) of nurses with a positive attitude toward geriatric nursing care. This was comparable with the other studies in Iran (45.7%) (23), Pakistan (51.8%) (24), Nepal (50.3%) (25), and west Shoa (45.7%) (13). However, this finding was lower compared to studies in Saudi Arabia (65%) (18), Bangladesh (63.8%) (26), and Ghana (84.5%) (21). The discrepancy might be due to, the difference in study settings and socio demographic characteristics of participants, lack of formal education and training about geriatric care, and the lack of special wards available for geriatric care in the study area. A previous study in Nigeria suggested special training and special wards are crucial for effective geriatric care, influencing nurses’ attitudes and requiring hospitals to have such facilities (27).

In this study level of education, years of work experience, and living experience with people aged 60 years or above were significantly associated with knowledge about geriatric nursing care. Respondents having a BSc degree or above were about two and half times more likely to have good knowledge about geriatric nursing care than respondents with a diploma in nursing. This is supported by previous studies conducted in Slovak (28), Bahir Dar Ethiopia (17), and West Shoa (13). This could be due to difference in the geriatric care contents in the course and also higher education levels may enable nurses to get access to a broader range of literature, improving their understanding of geriatric nursing care. This might be accomplished through training, continued professional development, and the addition of geriatric courses to the existing nursing curriculum.

The year of work experience is another factor that affected nurses’ knowledge about geriatric nursing care in this study. More experience nurses have more knowledgeable they are about geriatric nursing care. This finding is supported by a study in Bangladesh (26), a Study in Addis Ababa (11), and West Shoa (13). This could be because experienced nurses can enhance their knowledge through observation, practice, and sharing experiences with their colleagues. This suggests that working with experienced nurses can help to improve nurses’ knowledge and as do experienced nurses are required for effective geriatric nursing care. Compared to nurses who had not lived with older adult people, those who had lived with older adults were more likely to be knowledgeable about geriatric nursing care. This is consistent with the study in Zanzibar (29), Bahir Dar Ethiopia (17), and West Shoa (13). This could be due to the experience of giving care to older people at home which may have helped them to have better knowledge about geriatric nursing care.

In the current study, a higher level of education, years of work experience, living experience with older people, working room adequacy, and knowledge about geriatric nursing care were positively influenced the nurses’ attitude toward geriatric nursing care. Respondents with BSc degree or above in nursing have a more positive attitude compared to nurses with a diploma. This finding was consistent with studies conducted at public hospitals in West Shoa (13) and Addis Ababa (11). One explanation for this would be that nurses with a higher level of education have better knowledge about geriatric care which in turn might help them have a positive attitude toward geriatric nursing care.

Years of work experience in nursing was found to have a significant positive association with nurses’ attitude toward geriatric nursing care. Nurses with six and above years of experience were more likely to have a positive attitude than nurses with less than or equal to 5 years of experience in nursing. This is supported by studies in Bangladesh (26), and Addis Ababa (11). This might be because there were more opportunities to provide care for the older adult as a result of longer employment periods, which would have helped nurses gain better knowledge and experience, which in turn may have affected their attitudes. The previous study also suggested nurses who have experience in geriatric care, had a more positive attitude towards geriatric nursing care (30). In this study, Nurses ever lived with older people aged 60 years or above possessed a more positive attitude towards geriatric nursing care than those who did not live with older people. This could be due to frequent contact or exposure to older people. This finding is supported by previous Study which suggested that improved exposure to older people has a positive impact on attitude toward geriatric nursing care (22).

The current study also revealed nurses who perceived that the workspace/room was adequate during caregiving for older people were about two times more likely to have a positive attitude than nurses who did not work in an adequate space. This finding is supported by the study in Addis Ababa (11). This might be because hospitals lack dedicated spaces and medical equipment for caring for the older adult, which could result in unsuitable and crowded working conditions and affect nurses’ attitudes toward geriatric care. The previous studies also suggested that a work environment with inadequate resources had a negative influence on nurses’ attitude toward geriatric nursing care (27, 30). This study also found that Nurses having good knowledge about geriatric nursing care were three times more likely to have a positive attitude toward geriatric nursing care compared to nurses having poor knowledge about geriatric nursing care. This finding is supported by previous studies in Turkey (30), Israel (20), Taiwan (19), and Addis Ababa (11). This might be because nurses with good knowledge about geriatric care can better understand older people and their care which could contribute to having a positive attitude toward geriatric nursing care.

In this study, nurses’ marital status, age, and sex have no significant association with knowledge and attitude toward geriatric nursing care.

### 4.1 Strengths and limitations of the study

The current study’s strengths were that it assessed knowledge and attitudes toward geriatric care, which is care for a neglected age group in our country’s health system, and it was one of the first few studies in Ethiopia, and possibly the first in the study area, so the study will contribute much for the improvement of geriatric nursing care. This study covered both public and private hospitals, which may increase its generalizability. The current study also has limitations, the cross-sectional design of the study made it difficult to determine which occurred first among exposure and outcome, and the study may be vulnerable to recall bias because respondents were asked about the past.

## 5 Conclusion

According to this study, less than half of nurses demonstrated good knowledge and positive attitude toward geriatric nursing care. The higher level of education, the higher work experience, and having lived with older people aged 60 years or above were positively affected both the knowledge and attitude toward geriatric nursing care. Additionally, working in an adequate space and having knowledge about geriatric care positively influenced the attitude toward geriatric nursing care. Therefore, nursing schools, nursing associations, and hospitals should conduct training and consider the inclusion of geriatric nursing care contents in nursing education and in existing curriculum to enhance nurses’ knowledge about and attitude toward geriatric nursing care.

## Data availability statement

The raw data supporting the conclusions of this article will be made available by the authors, without undue reservation.

## Ethics statement

The studies involving humans were approved by Institutional Review Board (IRB) at college of Medicine and Health sciences Hawassa University. The studies were conducted in accordance with the local legislation and institutional requirements. The participants provided their written informed consent to participate in this study.

## Author contributions

WA: Conceptualization, Writing – original draft, Data curation, Formal analysis, Methodology, Writing – review & editing. AD: Writing – review & editing. BB: Writing – review & editing. MA: Writing – review & editing. TY: Writing – review & editing. DA: Writing – review & editing. TA: Writing – review & editing. FT: Writing – review & editing. TS: Writing – review & editing.
